# Alterations in Properties of Glutamatergic Transmission in the Temporal Cortex and Hippocampus Following Pilocarpine-Induced Acute Seizures in Wistar Rats

**DOI:** 10.3389/fncel.2017.00264

**Published:** 2017-08-31

**Authors:** Dmitry V. Amakhin, Sergey L. Malkin, Julia L. Ergina, Kirill A. Kryukov, Ekaterina A. Veniaminova, Olga E. Zubareva, Aleksey V. Zaitsev

**Affiliations:** ^1^Laboratory of Molecular Mechanisms of Neural Interactions, Sechenov Institute of Evolutionary Physiology and Biochemistry of the Russian Academy of Sciences Saint Petersburg, Russia; ^2^Federal Almazov North-West Medical Research Centre, Institute of Experimental Medicine Saint Petersburg, Russia

**Keywords:** NMDA receptors, hippocampus, temporal cortex, GluN2B-containing NMDA receptors, temporal lobe epilepsy, pyramidal neuron, animal model

## Abstract

Temporal lobe epilepsy (TLE) is the most common type of focal epilepsy in humans, and is often developed after an initial precipitating brain injury. This form of epilepsy is frequently resistant to pharmacological treatment; therefore, the prevention of TLE is the prospective approach to TLE therapy. The lithium-pilocarpine model in rats replicates some of the main features of TLE in human, including the pathogenic mechanisms of cell damage and epileptogenesis after a primary brain injury. In the present study, we investigated changes in the properties of glutamatergic transmission during the first 3 days after pilocarpine-induced acute seizures in Wistar rats (PILO-rats). Using RT-PCR and electrophysiological techniques, we compared the changes in the temporal cortex (TC) and hippocampus, brain areas differentially affected by seizures. On the first day, we found a transient increase in a ratio of α-amino-3-hydroxy-5-methyl-4-isoxazolepropionic acid (AMPA) and N-methyl d-aspartate (NMDA) receptors in the excitatory synaptic response in pyramidal neurons of the CA1 area of the dorsal hippocampus, but not in the TC. This was accompanied by an increase in the slope of input-output (I/O) curves for fEPSPs recorded in CA1, suggesting an enhanced excitability in AMPARs in this brain area. There was no difference in the AMPA/NMDA ratio in control rats on the third day. We also revealed the alterations in NMDA receptor subunit composition in PILO-rats. The GluN2B/GluN2A mRNA expression ratio increased in the dorsal hippocampus but did not change in the ventral hippocampus or the TC. The kinetics of NMDA-mediated evoked EPSCs in hippocampal neurons was slower in PILO-rats compared with control animals. Ifenprodil, a selective antagonist of GluN2B-containing NMDARs, diminished the area and amplitude of evoked EPSCs in CA1 pyramidal cells more efficiently in PILO-rats compared with control animals. These results demonstrate that PILO-induced seizures lead to more severe alterations in excitatory synaptic transmission in the dorsal hippocampus than in the TC. Seizures affect the relative contribution of AMPA and NMDA receptor conductances in the synaptic response and increase the proportion of GluN2B-containing NMDARs in CA1 pyramidal neurons. These alterations disturb normal circuitry functions in the hippocampus, may cause neuron damage, and may be one of the important pathogenic mechanisms of TLE.

## Introduction

Temporal lobe epilepsy (TLE) is the most common form of epilepsy in humans accompanied with hippocampal sclerosis (Curia et al., 2014). TLE is most frequently initiated by a primary brain damage that occurred during infancy (Mathern et al., 2002). This type of epilepsy is often resistant to pharmacological treatment, and prevention of hippocampal injury and epileptogenesis following a primary event might be a strategic innovative approach to epilepsy therapy. Unfortunately, the absence of clear data on the pathophysiological mechanisms leading to epilepsy does not allow any rational therapy (Curia et al., 2014). Therefore, it is essential to know in detail the pathogenic mechanisms of cell damage and epileptogenesis. It is suggested that the lithium-pilocarpine (PILO) model in rats reproduces many features of human TLE and it is often used experimentally to investigate the pathogenic mechanisms of TLE (Turski et al., 1983, 1989; Cavalheiro, 1995; Curia et al., 2008; Levesque et al., 2015; Malkin et al., 2016).

Due to the central function of α-amino-3-hydroxy-5-methylisoxazole-4-propionic acid (AMPA) and N-methyl d-aspartate (NMDA) glutamate receptors in excitatory neurotransmission (Traynelis et al., 2010), these receptors play a substantial role in the development of seizures; they are also involved in neuron damage and epileptogenesis (Kortenbruck et al., 2001; Porter et al., 2006; Borbely et al., 2009; Rogawski, 2011; Rajasekaran et al., 2012; Citraro et al., 2014; Zaitsev et al., 2015). Multiple alterations in glutamatergic neurotransmission following acute seizures have been reported, and in this study, we focused mostly on the properties of NMDAR-mediated synaptic currents.

The NMDARs are heteromeric assemblies of GluN1 and GluN2 (or GluN3) subunits; the GluN2 subunit is an critical factor in the pharmacological and biophysical characteristics of NMDARs and can influence NMDAR assembly, ion conductance, and synaptic plasticity (Traynelis et al., 2010; Shipton and Paulsen, 2014). The GluN2A subunit determines a higher channel open probability, noticeably faster kinetics than does the GluN2B subunit, which determines diminished open probability and slower channel kinetics (Erreger et al., 2005). An increasing number of experimental evidence suggests that GluN2A and GluN2B subunits play different roles in the modulation of neuron excitability and the development of TLE (Frasca et al., 2011; Di Maio et al., 2013; Loddenkemper et al., 2014; Chen et al., 2016; Kryukov et al., 2016). For example, the distinct role of GluN2A- and GluN2B-containing receptors in epileptogenesis following PILO injection has been demonstrated (Chen et al., 2007). It has also been shown that PILO injection alters NMDAR gene expression (Di Maio et al., 2011). The composition of the NMDAR subunit changes in chronic epilepsy, with synaptic GluN2B-containing receptors being predominant in the epileptic neurons of the hippocampus (Klatte et al., 2013). Phosphorylation of GluN2B Tyr1472 increases after prolonged febrile seizures and remains elevated for 7 days after that (Chen et al., 2016). Pentylenetetrazole (PTZ)-induced status epilepticus (SE) and PTZ-kindling also upregulate the expression of NMDAR GluN2B subunits in the hippocampus (Zhu et al., 2015; Postnikova et al., 2017). Our previous studies have demonstrated that PILO-induced SE disturbs the functional properties of NMDARs, and these alterations in synaptic transmission may contribute to the patogenesis of epilepsy and impairment of long-term hippocampal synaptic plasticity (Kryukov et al., 2016; Ivanov and Zaitsev, 2017).

In the present study, we investigated the after-effects of PILO-induced acute seizures on glutamatergic synaptic transmission in 3-week-old Wistar rats. We also compared the changes in the temporal cortex (TC) and the dorsal (DH) and ventral hippocampus (VH), brain areas differentially affected by seizures. We found that in hippocampal pyramidal neurons, the AMPA/NMDA ratio transiently increases after SE, as does the relative contribution of GluN2B-containing receptors in an NMDAR-mediated component of the glutamatergic response. None of these changes were detected in pyramidal neurons of the TC.

## Methods

### Animal preparation

The experiments were carried out on 3-week-old Wistar rats. All animal procedures followed the guidelines of the European Community Council Directive 86/609/EEC and were approved by the Animal Care and Use Committee of the Sechenov Institute of Evolutionary Physiology and Biochemistry of the Russian Academy of Sciences. On the first day of the experiment, LiCl (127 mg/kg; intraperitoneally (i.p.); Sigma–Aldrich, St. Louis, MO, USA) was administered to the rats (PILO-rats). On the second day, to reduce the peripheral muscarinic effects, methyl-scopolamine (1 mg/kg, i.p., Sigma–Aldrich) was administered, and 30–50 min later rats were injected with a single dose of muscarinic agonist pilocarpine dissolved in 0.9% NaCl (30 mg/kg, i. p.; Sigma–Aldrich) (Curia et al., 2008). In all rats included in the study, the pilocarpine-induced seizures lasted more than 30 min, usually 2–3 h (experimental status epilepticus, SE). Rats typically exhibited repeated head cloning movements, rearing and falling.

Control group consisted of rats injected with vehicle (RNA extraction, *N* = 7 rats; electrophysiology: patch-clamp recordings in TC, *N* = 9; in hippocampal area CA1, *N* = 7; field recordings in CA1, *N* = 6 rats). PILO-rats were sacrificed at the two time intervals following PILO administration: 24 h (RNA extraction, *N* = 7, electrophysiology: patch-clamp recordings in TC, *N* = 7; in CA1, *N* = 8; field recordings in CA1, *N* = 6); 3 days (RNA extraction, *N* = 7, electrophysiology: patch-clamp recordings in TC, *N* = 8; in CA1, *N* = 8; field potential recordings in CA1, *N* = 3). The brain of the same rat was typically used for both patch-clamp and field potential recordings.

### RNA extraction and real-time quantitative PCR

Rats were sacrificed and their brains were quickly removed and kept frozen at −80°C before RNA isolation. Brain region samples of temporal cortex (TC), dorsal (DH), and ventral parts of the hippocampus (VH) were dissected at −20°C using rat brain atlas (Paxinos and Watson, 2006). Extraction of total RNA was done with TRI Reagent (MRC, USA). Spectrophotometric method was employed to estimate the quality and quantity of RNA from each sample. Total RNA (2 μg) was converted to cDNA using oligo (dT) primer and M-MLV reverse transcriptase (Promega, Madison, WI, USA). Real-time quantitative polymerase chain reaction (qPCR) of GluN1 (GenBank NM_017010), GluN2a (GenBank NM_012573), GluN2b (GenBank NM_012574), and housekeeping gene Cyclophilin A (CycA, Ppia, GenBank NM_017101) was done with the TaqMan probes and the Bio-Rad CFX96 TouchTM Real-Time PCR Detection System (Bio-Rad Laboratories, Inc., USA). Primer and probe nucleotide sequences were synthesized by Alcor Bio Co (St. Petersburg, Russia) and shown in Table 1. Results were standardized to the housekeeping gene CycA and expressed as relative fold compared to control animals using the 2^−ΔΔCt^ method (Livak and Schmittgen, 2001).

**Table 1:** Nucleotide sequences of primers and probes.

**Gene**	**Forward primer (5′ →3′)**	**Revers primer (5′ →3′)**	**Probe (5′ →3′)**
GluN1	GTTCTTCCGCTCAGGCTTTG	AGGGAAACGTTCTGCTTCCA	CGGCATGCGCAAGGACAGCC
GluN2a	GCTACACACCCTGCACCAATT	CACCTGGTAACCTTCCTCAGTGA	TGGTCAATGTGACTTGGGATGGCAA
GluN2b	CCCAACATGCTCTCTCCCTTAA	CAGCTAGTCGGCTCTCTTGGTT	AGACGCCAAACCTCTAGGCGGACAG
CycA	AGGATTCATGTGCCAGGGTG	CTCAGTCTTGGCAGTGCAGA	ACACGCCATAATGGCACTGGTGGCA

### Slice preparation

The brain slice preparation was described previously in details (Kryukov et al., 2016; Malkin et al., 2016). A vibrating microtome (Microm HM 650 V; Microm; Germany) was used to cut horizontal 300-μm-thick slices that contained TC and hippocampus. Artificial cerebrospinal fluid (ACSF) with the following composition (in mM): 126 NaCl, 24 NaHCO_3_, 2.5 KCl, 2 CaCl_2_, 1.25 NaH_2_PO_4_, 1 MgSO_4_, and 10 dextrose were used through all steps. The ACSF was aerated with the gas mixture of 95% O_2_ and 5% CO_2_.

### Patch-clamp recordings

Recordings were performed at 30°C. Pyramidal neurons in deep layers of the TC or CA1-hippocampus were visualized using a Zeiss Axioscop 2 microscope (Zeiss; Oberkochen, Germany) equipped with differential interference contrast optics and a video camera (PointGrey Grasshopper3 GS3-U3-23S6M-C, FLIR Integrated Imaging Solutions Inc., USA). Patch electrodes (3–5 MΩ) were pulled from borosilicate glass capillaries with filaments (Sutter Instrument; Novato, CA, USA) on a P-1000 Micropipette Puller (Sutter Instrument). A cesium methanesulfonate-based solution (composition, in mM: 127 Cesium methanesulfonate, 10 NaCl, 5 EGTA, 10 HEPES, 6 QX314, 4 ATP-Mg, and 0.3 GTP; pH was adjusted to 7.25 with CsOH) was used in the experiments.

Whole-cell recordings were performed with Model 2400 (AM-Systems; Sequim, WA, USA) patch-clamp amplifier, and an NI USB-6343 A/D converter (National Instruments; Austin, TX, USA) using WinWCP5 software (SIPBS; Glasgow, UK). The data were filtered at 10 kHz and sampled at 20 kHz. In all cells included to the sample, access resistance was <15 MΩ and remained stable (≤20% increase) across the experiment. The liquid junction potential was estimated as described previously (Neher, 1992), and the holding potential was compensated offline for voltage-clamp recordings by subtracting 7 mV.

The synaptic responses were evoked extracellularly. The stimulating glass monopolar electrode was placed in the same layer of the TC as the recorded neuron and on Schaffer collaterals for CA1 pyramidal neuron at a distance of 100–200 μm in both cases. Excitatory postsynaptic currents (EPSCs) were recorded at various holding voltages from −107 to +33 mV with a step of 20 mV. We recorded AMPAR- and NMDAR-mediated synaptic currents in the presence of bicuculline (20 μM, Tocris Bioscience, Bristol, UK). Next, isolated NMDAR-mediated EPSCs were recorded in presence of DNQX (20 μM, Tocris Bioscience) and bicuculline (20 μM). These synaptic currents were completely abolished with AP-5 (50 μM, Tocris Bioscience). In some neurons isolated AMPAR-mediated synaptic currents were recorded in the presence of AP-5 (50 μM), MK-801 (18 μM), and bicuculline (20 μM).

### Estimation of AMPAR- and NMDAR-mediated conductances

To assess the relative impacts of AMPA and NMDA receptors during evoked response in each neuron, we implemented the mathematical approach described in our previous study (Amakhin et al., 2016).

First, we explored the I-V relationships for NMDAR- and AMPAR-mediated synaptic currents.

The voltage dependence of NMDA currents (peak amplitude vs. voltage) was investigated for each included neuron. The I-V curves were well approximated with the Boltzmann function (Jahr and Stevens, 1990; Harnett et al., 2015):

(1) $$I_{NMDA}\left(U\right)=\frac{g_{inf}}{1+\exp\left(\frac{V_{12}-U}{k}\right)}\left(U-V_{NMDA}\right),$$

where *g*_*inf*_ is the receptor conductance without the Mg^2+^ block as *U* approaches infinity; *V*_12_ and *k* determine the voltage dependence of the Mg^2+^ block of NMDARs; and *V*_*NMDA*_ is the reversal potential. g_*inf*_, *V*_*NMDA*_, *V*_12_, and *k* were allowed to vary during the approximation.

To introduce a conductance of NMDAR channels, Equation (1) was rewritten as following:

(2) $$I_{NMDA}(U)=g_{NMDA}\;f_{NMDA}\left(U\right),$$

where $g_{NMDA}={\frac{dI_{NMDA}}{dU}|}_{U\to \;+\infty }$ is the NMDAR conductance as the membrane voltage level approaches infinity, and *f*_*NMDA*_(*U*) is the voltage-dependent factor of Equation (1).

In some neurons, we investigated the I-V relationship of AMPAR-mediated current; it was found to be linear and to have the following form:

(3) $$I_{AMPA}\left(U\right)=g_{AMPA}\left(U-V_{AMPA}\right),$$

where *V*_*AMPA*_ is the reversal potential of AMPA current and *g*_*AMPA*_ is the conductance of postsynaptic AMPARs. For each recorded cell the assumed value of *V*_*AMPA*_ was set equal to the experimentally obtained value of *V*_*NMDA*_.

Next, to estimate the AMPAR- and NMDAR-mediated conductances we used the eEPSCs recorded at various holding potentials from −107 to +33 mV with a step of 20 mV. The I-V curves were obtained every 300 μs. AMPAR- and NMDAR-mediated conductances were estimated by fitting each I-V curve obtained from the set of responses with a two-parameter function of the total current (*I*_*total*_):

(4) $$\begin{array}{l} I_{total}\left(U;g_{AMPA},\;g_{NMDA}\right)=\;g_{NMDA\;}f_{NMDA}\left(U\right)\\ \;+g_{AMPA}\left(U-V_{AMPA}\right), \end{array}$$

where *f*_*NMDA*_(*U*) is the function from Equation (2). Conductances were allowed to vary in a positive range only. The least-square method was utilized to find a good estimate of conductances using Wolfram Mathematica 10 (Champaign, IL, USA).

To compare the time course of NMDAR-conductance before and after the block of AMPARs, we estimated the latter by fitting the I-V curves obtained from the set of responses in the presence of DNQX with a single parameter function of the total current:

(5) $$I_{total}\left(U;\;g_{NMDA}\right)=g_{NMDA\;}f_{NMDA}\left(U\right)\;$$

### Analysis of properties of the NMDAR-mediated evoked EPSCs

The contribution of GluN2B-containing NMDARs was estimated using a selective antagonist of GluN2B-containing NMDARs, ifenprodil (3 μM, Tocris Bioscience). Pharmacologically isolated NMDAR-mediated eEPSCs were recorded in cortical and CA1 pyramidal neurons at +33 mV before and after 12 min of exposure to ifenprodil. A relative block of NMDAR-mediated current was calculated:

(6) $$\text{Block}=100\%\times \frac{A_{baseline}-A_{ifenprodil}}{A_{baseline}},$$

where *A*_*baseline*_ and *A*_*ifenprodil*_ are the average eEPSC amplitudes (pA) or areas (pC) before (at the baseline) and 12 min after ifenprodil administration, respectively.

Kinetics of NMDAR-mediated eEPSCs at +33 mV was estimated using non-linear regression analysis on the decay phase (10–90%; Szinyei et al., 2003; Gonzalez-Burgos et al., 2008). We utilized both biexponential (Equations 7, 8) and monoexponential functions (Equation 9).

(7) $$\begin{array}{l} I\left(t;A_{fast},\tau_{fast},A_{slow},\tau_{slow}\right)=A_{fast}\;*\;\exp\left(-\frac{\text{t}}{\tau_{fast}}\right)\\ \;+A_{slow}\;*\;\exp\left(-\frac{\text{t}}{\tau_{slow}}\right) \end{array}$$

or

(8) $$I\left(t;A_{fast},A_{slow}\right)=A_{fast}\;*\;\exp\left(-\frac{\text{t}}{60}\right)+A_{slow}\;*\;\exp\left(-\frac{\text{t}}{300}\right),$$

where *A*_*fast*_ and τ_*fast*_ are the amplitude and time constant of fast decaying component; *A*_*slow*_ and τ_*slow*_ are the amplitude and time constant of slow decaying component.

(9) $$I\left(t;A,\tau \right)=A\;*\;\exp\left(-\frac{\text{t}}{\tau }\right),$$

where *A* and τ are the amplitude and time constant of the response.

The Akaike information criterion (AIC) was used as a measure of the relative accuracy of the approximation.

The weighted time constants were calculated using the following formula:

(10) $$\tau_{weighted}=\frac{\tau_{fast}\;*\;\;A_{fast}+\tau_{slow}\;*\;A_{slow}}{A_{fast}+A_{slow}},$$

Where *A*_*fast*_, τ_*fast*_, *A*_*slow*_ and τ_*slow*_ were obtained by fitting the decay phase of the response with Equation (7).

The relative contribution of the slowly decaying component was calculated as following:

(11) $$RC_{slow}=\frac{A_{slow}}{A_{fast}+A_{slow}}$$

where *A*_*fast*_ and *A*_*slow*_ are the amplitudes of the fast and slow decaying components, respectively, which were obtained by fitting the decay phase of the response with Equation (8).

### Recording and analysis of field excitatory postsynaptic potentials

Slices were transferred to the recording chamber. The field excitatory postsynaptic potentials (fEPSP) were recorded in *stratum radiatum* of CA1 area with a glass microelectrode filled with ACSF (0.2–1.0 MΩ). Field potentials were registered with Model 1800 amplifier (AM-Systems), and an NI USB-6211 A/D converter (National Instruments) using WinWCP5 software (SIPBS). A bipolar twisted stimulating electrode made of insulated nichrome wire (0.1 mm in diameter) was mounted in *stratum radiatum*, in the region of the Schaffer collateral pathway, between areas CA2 and CA1 of the rat hippocampus. The stimulation was performed with rectangular pulses (duration, 0.1 ms) every 20 s using constant current stimulus isolator A365R (WPI, Sarasota, FL, USA).

The dependence of field response amplitude on stimulus strength was determined by increasing the current intensity from 25 to 250 μA at a pace of 25 μA, and the amplitudes of the presynaptic fiber spike (fiber volley, FV) and fEPSPs were measured. Recordings were analyzed using Clampfit 10.0 (Molecular Devices Corporation, USA). The plot of the fEPSP amplitude against current intensity was fitted with a sigmoidal Gompertz function (Equation 12) using OriginPro 8 (OriginLab Corporation, Northampton, MA, USA):

(12) $$y=ae^{-e^{\left(-k\left(x-x_{c}\right)\right)}},$$

where *a* is an asymptote of maximum fEPSP amplitude (in mV); *e* is Euler's Number (*e* = 2.71828…); *k* and *x*_*c*_ are positive numbers describing the shape of the curve; *x*_*c*_ is a value of current (in μA) at which the maximum slope of the curve is observed; *ak*/*e* is a maximum slope of the curve (in mV/μA).

We use the predicted values from that curve for each slice to add to group data.

### Statistics

Statistical analysis and plotting were conducted using SigmaPlot 12.5 software (SYSTAT Software; San Jose, CA, USA). Dixon's Q test (at 90% confidence) was used for rejecting the outliers. Normality of sample data was evaluated with the Kolmogorov-Smirnov test. Equality of variance was assessed with the Levene median test. For data that had the normal distribution and passed equal variance test, we applied Student's *t*-test and one-way ANOVA following with Dunnett's *post hoc* test. For data that did not pass the normality test, we used Kruskal-Wallis one-way ANOVA on Ranks test. The results are expressed as a mean ± standard error of the mean (stardard deviation).

## Results

### Changes of relative contribution of AMPARs and NMDARs in synaptic response following PILO-induced SE

**Figure 1:**
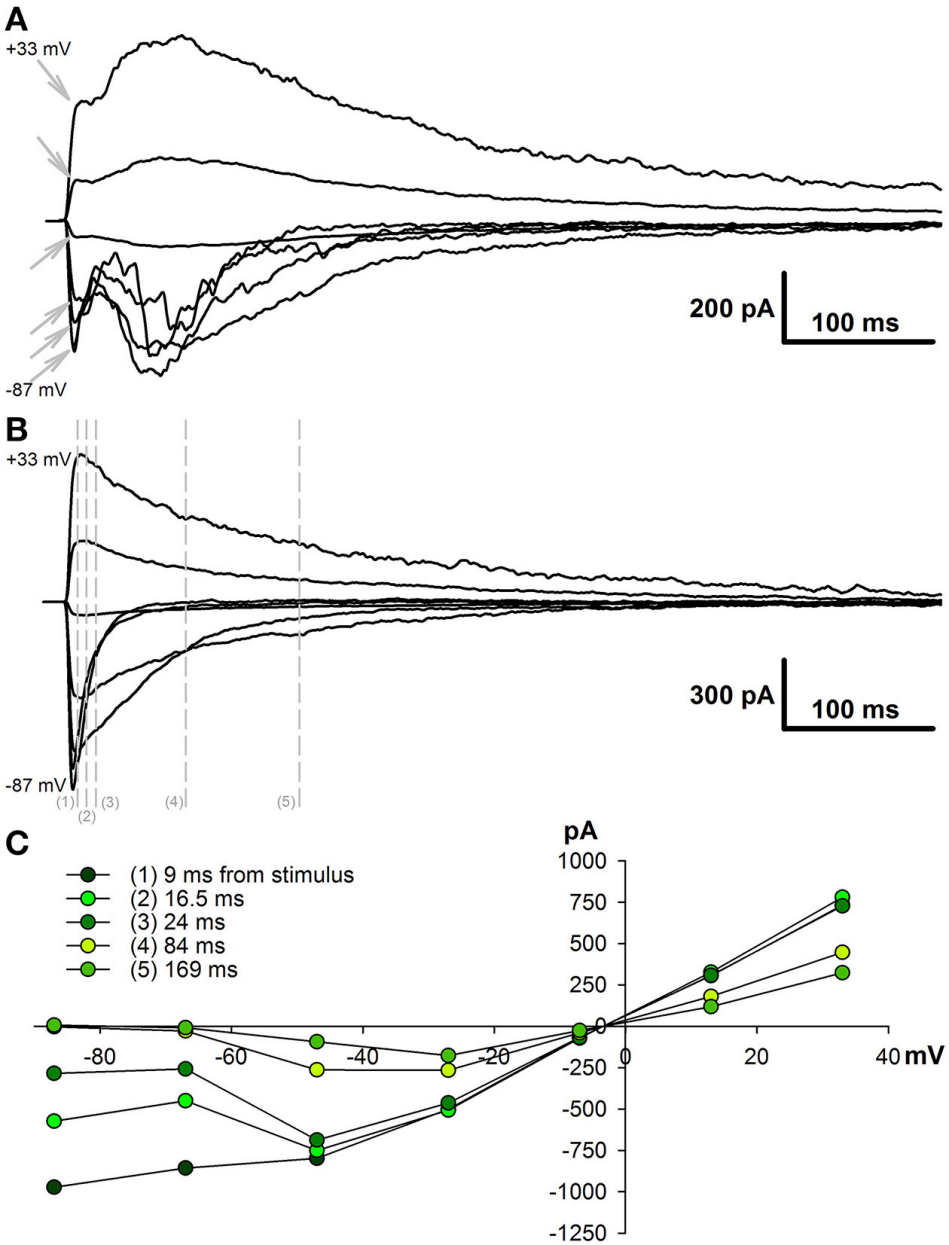
Evoked EPSCs in pyramidal cells of TC and hippocampus. **(A)** A representative example of evoked EPSCs recorded in the TC pyramidal cell in control rat at different holding voltages (from +33 to −87 mV with 20 mV increment). Stimulus artifacts were digitally subtracted. Note that the responses have clearly distinct monosynaptic peaks (marked with gray arrows). **(B)** Evoked EPSCs recorded in the CA1 pyramidal cell in control rat. Note the absence of polysynaptic components of the response. Vertical lines indicate the time points at which I–V relationships are shown in C. **(C)** I–V relationships obtained at different time point after stimulation. Note that at the beginning (9 ms from the stimulus) I-V is almost linear and is similar to AMPA channel I-V curve, later it resembles curve for the conductance elicited by NMDA receptors.

First, we evaluated whether the relative contribution of AMPAR- and NMDAR-mediated synaptic conductances changed following SE in the pyramidal neurons of the two brain regions (TC and hippocampus) involved in TLE. For this purpose, we evoked EPSCs in cortical and CA1 pyramidal neurons in slices obtained from control animals and PILO-rats. We recorded EPSCs at various holding potentials (Figures 1A,B). In the presence of bicuculline, the responses of cortical neurons frequently had polysynaptic components, even if we used the minimum stimulation. In contrast, the polysynaptic responses were rare in hippocampal neurons. We included in the analysis only the responses with a clearly distinct monosynaptic peak (Figure 1A, monosynaptic peaks are marked with arrows).

**Figure 2:**
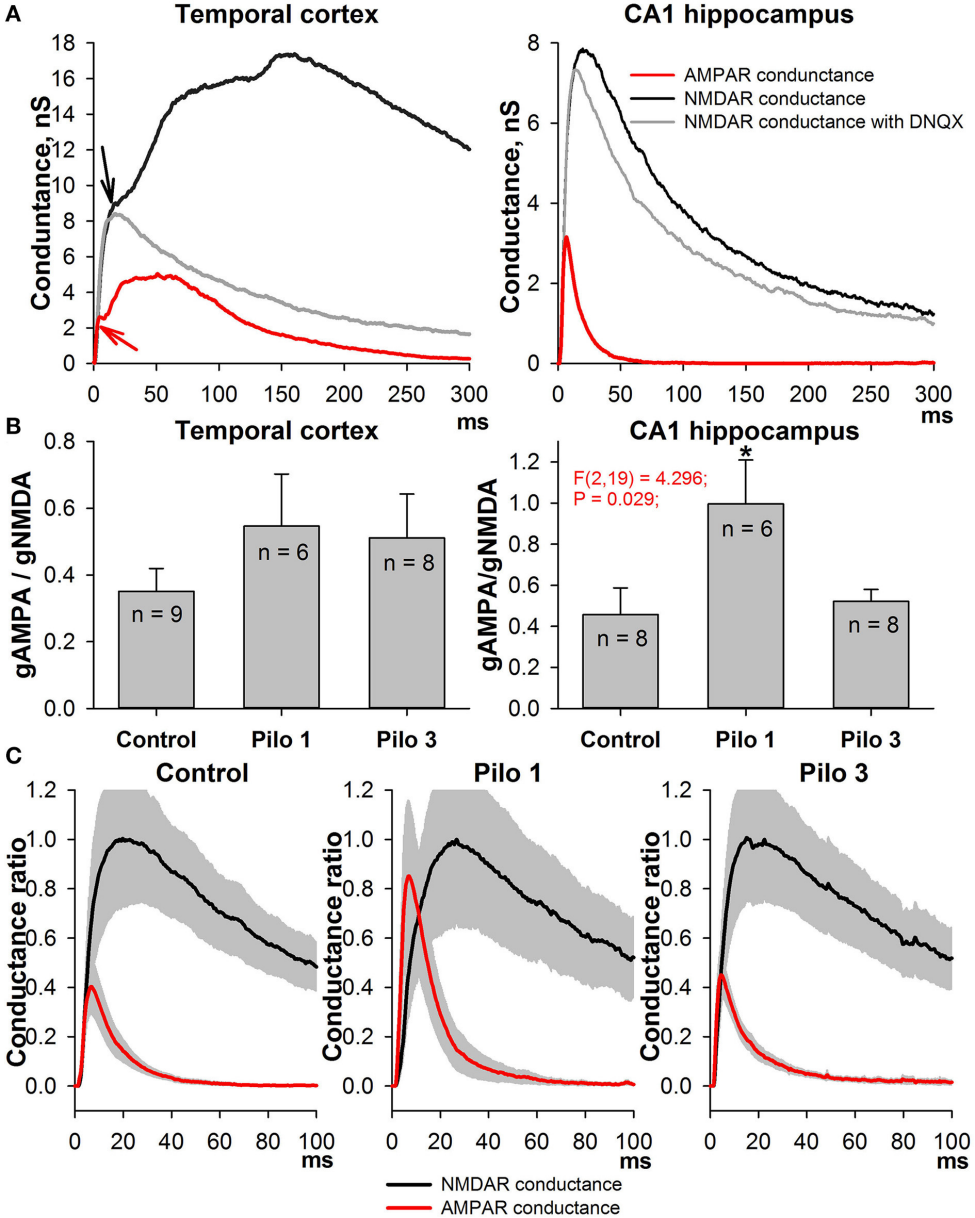
Changes in AMPAR/NMDAR conductance ratios in PILO-rats. **(A)** The estimated conductances as functions of time for TC neurons (left, an average of 9 cells) and CA1 neurons (right, an average of 8 cells). Arrows on the right plot indicate the monosynaptic peaks of AMPAR- and NMDAR-mediated conductances, which were used to assess the ratios on **(B)**. The red line represents the time course of AMPAR conductance; the black line is calculated NMDAR conductance during the evoked EPSCs; and the gray line—NMDAR conductance after the block of AMPARs. **(B)** Peak AMPAR/NMDAR conductance ratios for TC (left) and CA1 neurons of control (Control) and PILO-rats sacrificed at the following time intervals after PILO administration: Pilo 1 – 24 h, Pilo 3 – 3 days. An asterisk (*) indicates significant difference (*p* < 0.05) with the control group (Dunnett's *post hoc* test, multiple comparisons vs. control group). **(C)** The time course of AMPAR- and NMDAR-mediated conductances during the evoked response in the hippocampal pyramidal neurons in control and PILO-rats. A gray area shows the standard error.

To estimate the AMPAR- and NMDAR-mediated synaptic conductances, we built a set of I-V curves at various time points from the stimulus (Figure 1C) and fitted them with Equation (4) using the conductances *g*_*AMPA*_ and *g*_*NMDA*_ as coefficients. Next, we plotted the calculated conductances as functions of time for cortical and hippocampal neurons (Figure 2A). The obtained functions of time indicated that NMDAR-mediated conductance peaks with a delay of 16.9 ± 1.6 (7.5) ms (*n* = 22) after the peak of AMPAR-mediated conductance.

To confirm our calculations experimentally, we used DNQX, a selective AMPAR antagonist. The DNQX completely abolishes the polysynaptic components of the response in the cortical pyramidal neurons, but it does not affect the properties of the monosynaptic NMDAR-mediated component. No significant differences (paired *t*-test, *p* > 0.05) were found between the time and amplitude of the peaks of the monosynaptic NMDAR-mediated conductance in eEPSCs mediated by both AMPARs and NMDARs (black line) and in pharmacologically isolated NMDAR-mediated eEPSCs (gray line). The latter remained true for hippocampal neurons: the estimated peaks of NMDAR-mediated conductance within the responses of both glutamate receptors and only NMDARs were the same (paired *t*-test, *p* > 0.05).

Next, we calculated the ratio of monosynaptic peak conductances (AMPA/NMDA ratio) to assess the relative contribution of AMPAR- and NMDAR-mediated synaptic conductance during the evoked response. We found that, in control animals, the AMPA/NMDA ratio ranges from 0.3 to 0.6 in both the cortical and the hippocampal neurons (Figure 2B); no significant difference was observed between these areas (paired *t*-test, *p* > 0.05). On the day after the PILO-induced SE, the ratio significantly increased in the hippocampal neurons and returned to its control value by the third day [one-way ANOVA, *F*_(2, 19)_ = 4.30, *p* = 0.03, followed by Dunnett's *post hoc* test, Figure 2B]. No changes in the AMPA/NMDA ratio following SE were found in cortical neurons [one-way ANOVA, *F*_(2, 20)_ = 0.844, *p* = 0.45].

In addition, we compared the time course of AMPAR- and NMDAR-mediated conductances during the evoked response in the hippocampal pyramidal neurons in control and PILO-rats (Figure 2C). We analyzed the overall dynamics of estimated conductances (absolute peak amplitudes, rise times, half-widths, decay times, and positions of the peaks). However, no other significant changes were detected in the measured parameters following SE.

**Figure 3:**
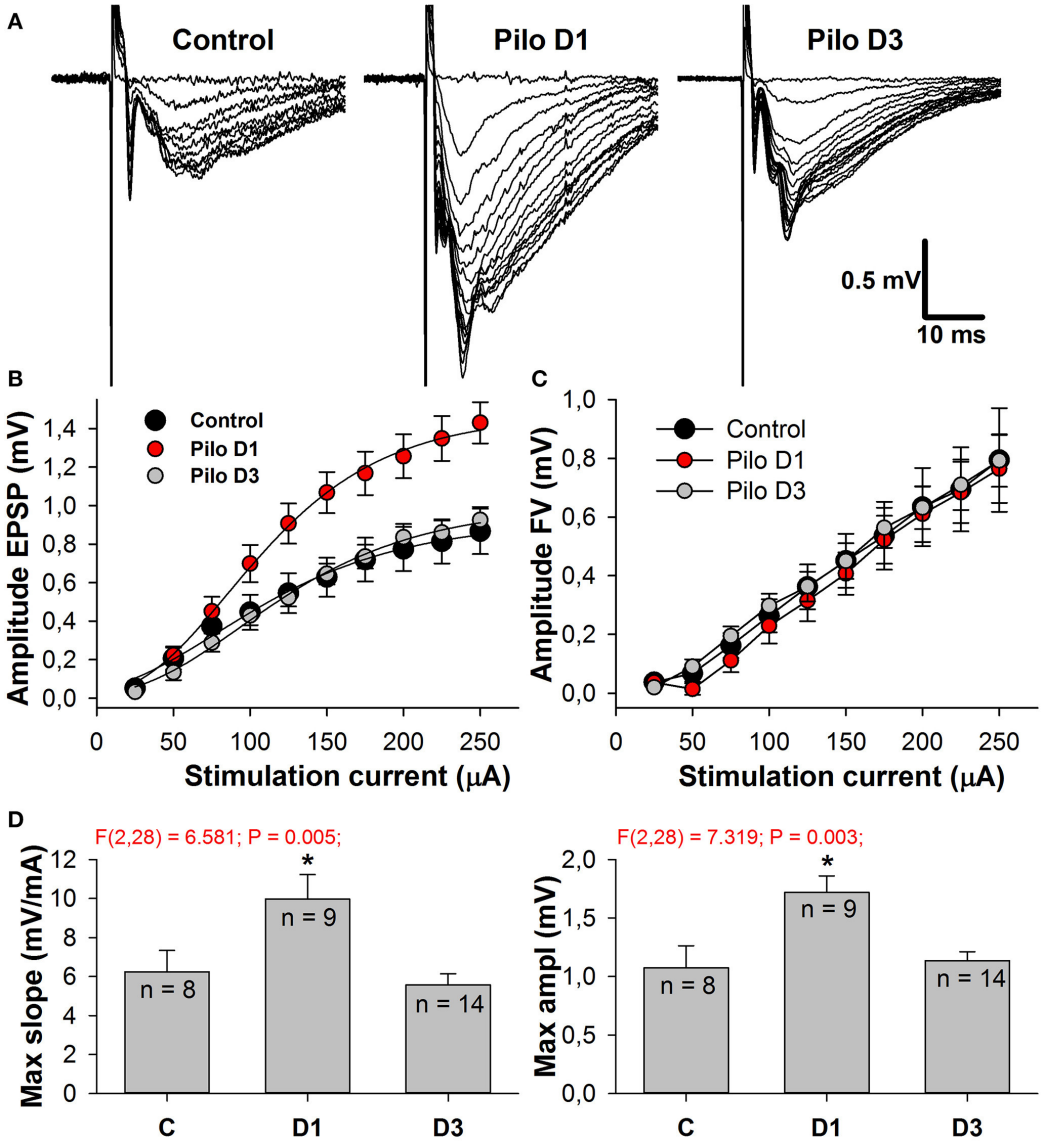
Amplitudes of fEPSPs are enhanced in PILO-rats. **(A)** Representative examples of fEPSPs recorded in the CA1 hippocampus of control and PILO-rats in 1 day (Pilo D1) and 3 days (Pilo D3) after administration of the drug. **(B)** and **(C)** Input-output (I/O) curves for the fEPSPs **(B)** and fiber volleys **(C)**. **(D)** Bar graphs showing maximum slope of I/O curves for the fEPSPs (left) and predicted maximum amplitude of fEPSPs (right). An asterisk (*) indicates significant difference (*p* < 0.05) with the control group (Dunnett's *post hoc* test, multiple comparisons vs. control group).

This alteration in the AMPA/NMDA ratio might be attributed equally to the augmentation of AMPAR-mediated conductance and to the reduction of NMDAR-mediated conductance. To test these possibilities, we built the input-output (I/O) curves for the fEPSP recorded in CA1 (Figure 3). A stimulating electrode was placed on the slice to activate the Schaffer collaterals. Because the contribution of NMDARs to fEPSP is relatively small, any differences in I/O curves are dependent specifically on AMPAR-mediated input (Woolley et al., 1997). We found that the slope of the I/O curves and the predicted maximum amplitude of fEPSPs increase on the first day after SE [Figures 3B,D, one-way ANOVA followed by Dunnett's *post hoc* test; slope: *F*_(2, 28)_ = 6.58, *p* < 0.01, control −6.25 ± 1.01 (2.86) mV/μA, *n* = 8 vs. Pilo 1D - 9.97 ± 1.26 (3.78) mV/μA, *n* = 9; amplitude: *F*_(2, 28)_ = 7.32, *p* < 0.01, control - 1.07 ± 0.19 (0.54) vs. Pilo 1D - 1.72 ± 0.14 (0.42) mV] and return to control value on the third day [slope: 5.56 ± 0.58 (2.17) mV/μA; amplitude: 1.14 ± 0.08 (0.30) mV, *n* = 14]. The change in the slope of the I/O curves may be due to the increased presynaptic activation of the fibers. To test this possibility, we investigated the relationship between the stimulus strength and the amplitude of FV (Figure 3C). FV represents a summation of many spikes that arrive at the CA1 region after Schaffer collateral stimulation. We did not find any difference between the curves obtained in the control and experimental groups [the slope of linear approximation was analyzed with one-way ANOVA: *F*_(2, 26)_ = 0.35, *p* = 0.7]. These results suggest that the excitability of Schaffer's collaterals does not change following PILO-induced SE and, therefore, a steeper slope in the I/O curve indicates that AMPAR conductance increases on the first day following SE.

### Changes in expression level of NMDA receptor subunit mRNAs following PILO-induced SE

**Figure 4:**
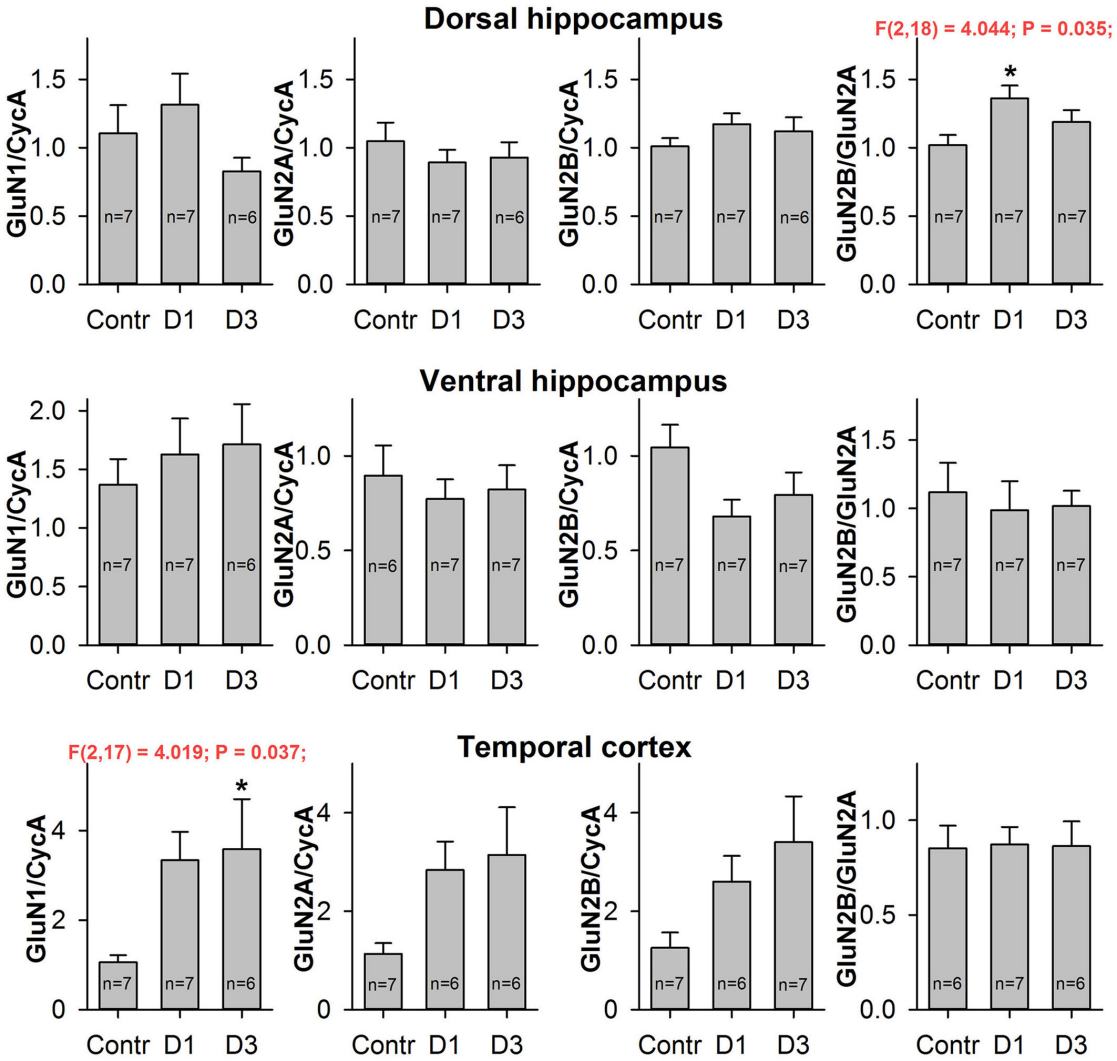
Quantitative real-time PCR analysis of NMDAR subunit mRNA. The mRNA expression of GluN1 subunits (first column), GluN2A subunits (second column), GluN2B subunits (third column) and GluN2B/GluN2A expression ratio (fourth column) in dorsal hippocampus (top), ventral hippocampus (middle), and temporal cortex (bottom) of control (Contr) and PILO- rats sacrificed in 1D – 24 h, 3D – 3 days after PILO administration. For datasets with detected significant differences, the corresponding one-way ANOVA results are presented in the chart. Asterisks (*) indicate significant difference (*p* < 0.05) from the control group (Dunnett's *post hoc* test, multiple comparisons vs. control group).

To estimate the effects of seizures on the expression level of NMDAR subunit mRNAs, we performed qPCR for GluN1, GluN2A, and GluN2B subunits in two hippocampal areas (DH and VH) and in the TC of the control and PILO-rats, 1 and 3 days after PILO administration. We observed that PILO-induced SE differentially affected hippocampal and cortical mRNA expression (Figure 4). Pilocarpine-induced SE increases the mRNA level of the GluN1 subunit in the TC [one-way ANOVA, *F*_(2, 17)_ = 4.02; *p* < 0.05], but not in the hippocampus [DH: *F*_(2, 17)_ = 1.53; *p* = 0.25; VH: *F*_(2, 17)_ = 0.38; *p* = 0.69]. The increase of GluN1 expression in the TC reaches a significant level on the third day after SE (Dunnett's *post hoc* test, *p* < 0.05). Because GluN1 is an obligate subunit, its overexpression may indicate an increase in the total number of NMDARs in the TC on the third day after SE. In line with this observation are changes in GluN2A and GluN2B expression, which had similar tendencies to those of GluN1 [one-way ANOVA, *F*_(2, 16)_ = 3.092; *p* = 0.07; and *F*_(2, 17)_ = 2.907; *p* = 0.08, respectively].

We did not find any changes in the mRNA expression of the NMDAR subunits examined in either the DH or the VH. However, we found a growth in the GluN2B/GluN2A expression ratio in the DH [one-way ANOVA, *F*_(2, 18)_ = 4.04; *p* < 0.05], which reached a significant level on the first day after SE (Dunnett's *post hoc* test, *p* < 0.05). This observation suggests that changes in the subunit composition of NMDARs are very likely.

### Changes in kinetics of NMDAR-mediated EPSCs following SE

**Figure 5:**
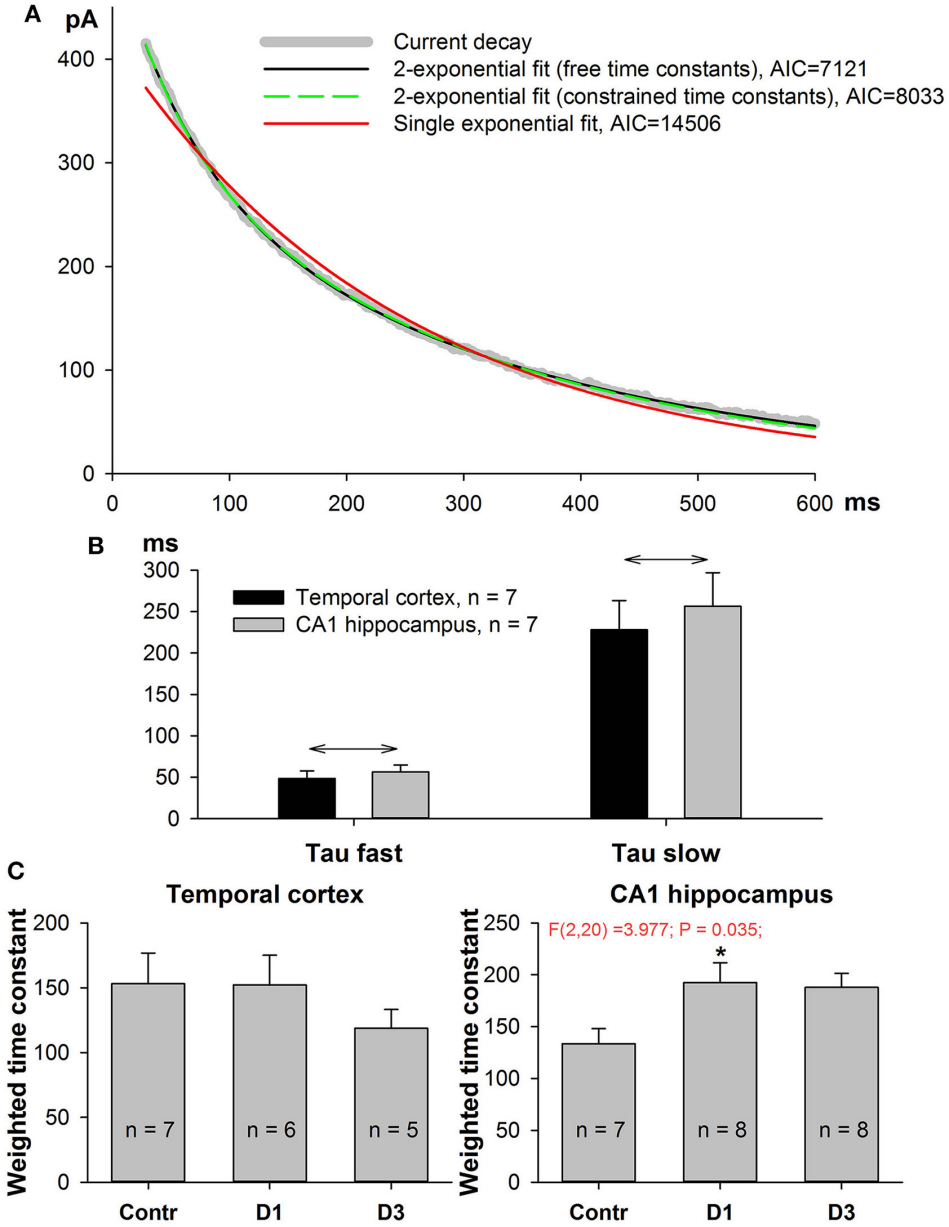
Kinetics of NMDAR-mediated evoked EPSC slow down in PILO-rats. **(A)** A representative 90–10% decay phase of NMDAR-mediated evoked EPSC fitted with various functions. The black line is a double exponential fit with Equation (7) (time constant were allowed to vary during approximation, resulting in τ_*fast*_ = 56 ms, τ_*slow*_ = 345 ms). Green dashed line represent a double exponential fit with Equation (9) with the fixed time constants, τ_*fast*_ = 60 ms, τ_*slow*_ = 300 ms. An Equation (9) with constrained time constants produces an approximation which is indistinguishable from the one produced by an Equation (7) and produces a much better fit (smaller AIC) than monoexponential function [Equation (8), red line]. **(B)** In both brain regions the average fast and slow time constants obtained by fitting with Equation (7) are significantly different. Asterisks (*) indicate significant difference (*p* < 0.05) between the fast and slow time constants (paired *t*-test). **(C)** In both brain regions, the partial block of evoked EPSC by ifenprodil does not significantly affect the time constants (paired *t*-test).

The GluN2A-NMDARs exhibit higher channel open probability and faster kinetics than the GluN2B-containing receptors (Erreger et al., 2005; Sun et al., 2017). Therefore, changes in NMDAR subunit composition might be detected by the kinetic properties of the NMDAR synaptic current. We performed non-linear regression analysis on the decay phase (90–10%) of the NMDAR-mediated current at +33 mV as described in the Methods section. The current decays were better fitted with the biexponential function Equation (7) than with the mono-exponential one (Equation 9, Figure 5A); therefore, in subsequent analyses, we utilized only the parameters obtained using the bi-exponential fit. The average τ_*fast*_ was about 60 ms and the average τ_*slow*_ was about 300 ms (Figure 5B). No significant differences were found between TC- and CA1-neurons in the corresponding time constants (*t*-test, *p* > 0.05 for both fast and slow time constants). Next, we calculated the weighted decay time constant using Equation (10) and analyzed its changes after PILO-induced SE (Figure 5C). No significant changes were detected in TC neurons [one-way ANOVA, *F*_(2, 15)_ = 0.72, *p* = 0.50], while in CA1 pyramidal neurons, an increase in the weighted time constant was detected [one-way ANOVA, *F*_(2, 20)_ = 3.98, *p* < 0.05]. The increase reached a significant level on the first day after SE (Dunnett's *post hoc* test, *p* < 0.05), indicating that the decay phase slowed down after SE. These data are in line with the possibility that the percentage of GluN2B-containing receptors in hippocampal synapses increases following SE.

### The effects of ifenprodil on NMDAR-mediated EPSCs

**Figure 6:**
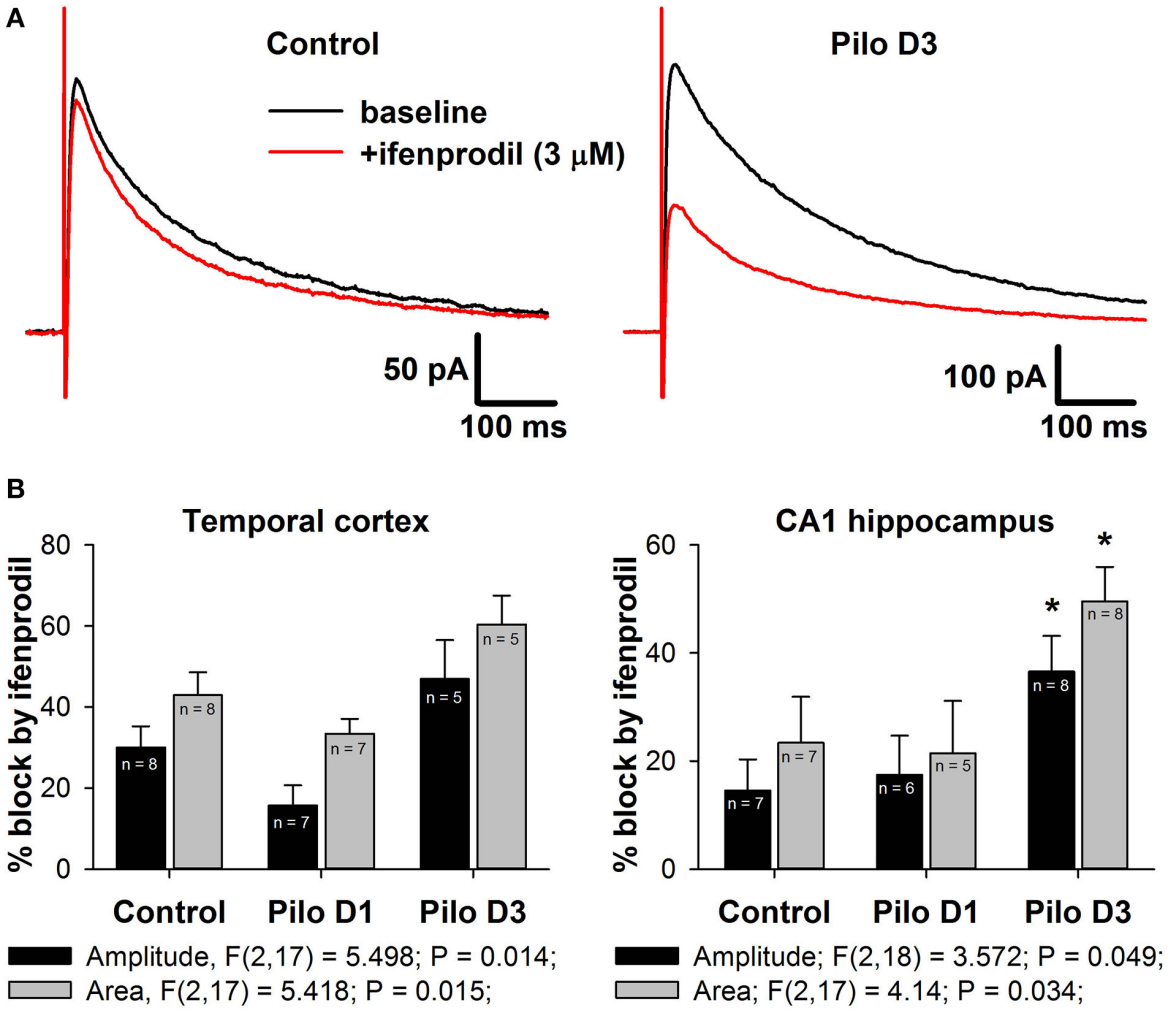
Effects of a selective GluN2B-containg NMDAR antagonist ifenprodil on evoked NMDAR-mediated synaptic currents. **(A)** Representative examples of recordings in hippocampal pyramidal neurons of control and PILO-rats. Black trace—the averaged initial synaptic response, red trace—the averaged synaptic response after application of ifenprodil. **(B)** Diagrams illustrating the block of current peak amplitude (black bars) and response area (gray bars) of NMDAR-mediated synaptic currents in pyramidal cells of control and PILO-rats. One-way ANOVA results are presented below the charts. Asterisks indicate significant difference (*p* < 0.05) from the control group (multiple comparisons vs. control group, Dunnett's test).

Next, we tested this possibility by investigating the effect of a selective GluN2B-containing NMDAR antagonist, ifenprodil (3 μM), on the evoked NMDAR-mediated EPSCs in the TC and DH (Figure 6A). In the control, application of ifenprodil decreased both eEPSC amplitude (by 30% in cortical and 15% in hippocampal pyramidal neurons, Figure 6B, black bars) and area (by 45% in the TC and 25% in the DH, Figure 4B, gray bars). In both brain areas, the magnitude of the ifenprodil block changed following PILO-induced SE [one-way ANOVA, amplitude: TC, *F*_(2, 17)_ = 5.50; *p* < 0.05; DH, *F*_(2, 18)_ = 3.57; *p* < 0.05; area: TC, *F*_(2, 17)_ = 5.42; *p* < 0.05; DH, *F*_(2, 17)_ = 4.14; *p* < 0.05; Figure 6B], but *post hoc* analysis confirmed changes only in the hippocampus. The observed increase in ifenprodil efficiency is consistent with the increase in the GluN2B/GluN2A expression ratio in synapses following SE.

**Figure 7:**
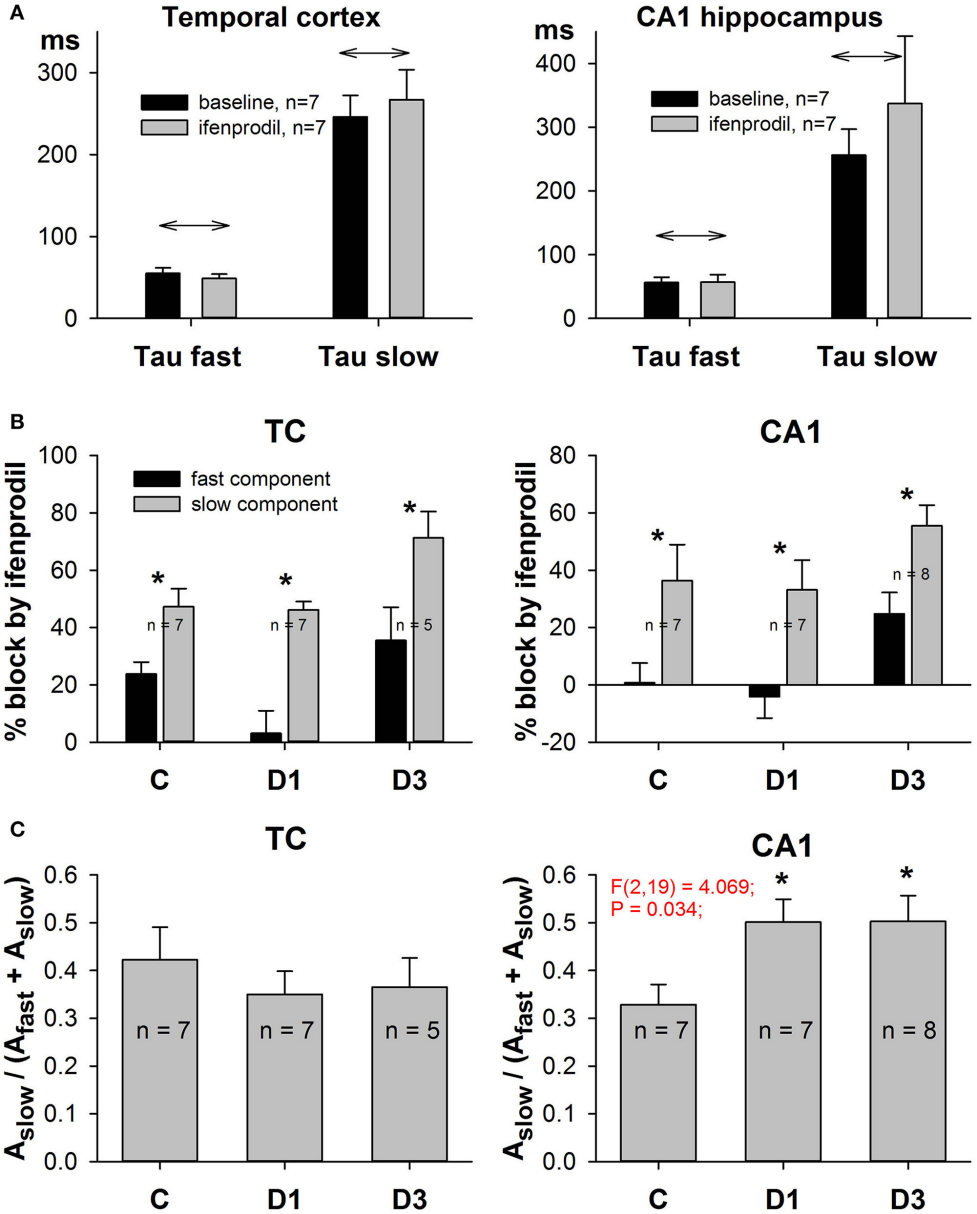
Effects of ifenprodil on fast and slow components of NMDAR-mediated EPSCs. **(A)** Ifenprodil does not change the values of the fast and slow time constants. **(B)** Ifenprodil differentially affects the fast and slow time constants, obtained by fitting the decay phase of EPSC with Equation (9). In TC and CA1 in control and PILO rats ifenprodil blocked the fast component of EPSC to a greater extent than the slow component. Asterisks (*) indicate significant difference (*p* < 0.05) between the relative block of fast and slow components (paired *t*-test). **(C)** The relative contribution of the slow ifenprodil-sensitive component to the decay of EPSC changes after SE. Asterisks (*) indicate significant difference (*p* < 0.05) from the control group (multiple comparisons vs. control group, Dunnett's test).

Next, we analyzed the effect of ifenprodil on the NMDAR-mediated current decay kinetics in more detail. We found that ifenprodil did not change the values of the fast and slow time constants (Figure 7A, paired *t*-test, *p* > 0.05 in all cases), but did change the impact of the fast and slow components. Thus, to reduce the number of parameters in a fit and to force all the potential changes in subunit composition to display themselves as changes in amplitudes of the slow or fast time constants, we utilized Equation (8) for fitting the decays of NMDAR-mediated currents. We found that ifenprodil reduces the amplitude of the slowly decaying component more than it reduces the amplitude of the rapidly decaying component (paired *t*-test, *p* < 0.05 in all cases, Figure 7B). This finding indicates that we can assess the relative contribution of GluN2B-containing NMDARs in synaptic response by calculating the proportion of slower decaying components with Equation (10). The results of this estimation are presented in Figure 7C. No significant changes in the relative contribution of the slowly decaying (and more ifenprodil-sensitive) component were found in the TC [one-way ANOVA, *F*_(2, 17)_ = 0.43, *p* = 0.66], while in CA1, we detected an increase [one-way ANOVA, *F*_(2, 19)_ = 4.07; *p* = 0.03]. The increase reached significant levels on the first and third day after PILO-induced SE (Dunnett's *post hoc* test, *p* < 0.05 in both cases).

Taken together, these facts indicate that SE increases the proportion of synaptic GluN2B-containing NMDARs in hippocampal pyramidal neurons.

## Discussion

TLE is frequently initiated by a primary brain damage during infancy (Mathern et al., 2002), therefore, we performed present study using the 3-week-old rats. We investigated changes during the first 3 days after seizures, the beginning of the latent period, which is considered as period of epileptogenesis (Curia et al., 2008). The major finding of this study is that PILO-induced SE leads to more severe alterations in excitatory synaptic transmission in the hippocampus than in the TC. In the hippocampus, it affects both the relative contribution of NMDA-receptor conductance in synaptic response and the subunit composition of NMDARs. Status epilepticus increases the proportion of GluN2B-containing NMDARs in synapses, which may affect normal circuitry functions.

### Changes in the AMPA/NMDA ratio following SE

We have demonstrated that in CA1 pyramidal neurons, the AMPA/NMDA peak conductance ratio transiently increases on the first day after PILO-induced SE. To estimate the conduction ratio we used a modification of the most basic method (Borg-Graham et al., 1998; Anderson et al., 2000; Monier et al., 2008; Ziburkus et al., 2013), which implies intracellular measurements of currents at various levels of membrane potential. The primary method assumes only two types of synaptic input (excitatory and inhibitory), known reversal potentials for both excitatory and inhibitory currents, and synaptic currents linearly dependent on voltage. Algebraic calculations provide estimates for the corresponding conductances if the synaptic responses are recorded at two or more different membrane potentials. However, the primary method cannot be utilized to distinguish between NMDAR- and AMPAR-mediated synaptic inputs as they have close reversal potentials and different form of I-V curves. To find the conductances we have to know the mathematical description of I-V curves for each current. We used a linear function for AMPAR-mediated current and the Boltzmann function (Jahr and Stevens, 1990; Harnett et al., 2015) for NMDAR-mediated current. The main difference of this approach from the one utilized in our previous study (Amakhin et al., 2016) is that reference I-V relationship for NMDAR-mediated current (*f*_*NMDA*_(*U*) in Equations (1) and (2) was not pre-recorded in a separate set of experiments but was obtained for every recorded cell individually, thus allowing us to account for slight variations of individual shape of I-V relationships in order to perform a more accurate estimation of synaptic conductances. I-V curves for isolated NMDAR-mediated currents were obtained in presence of inhibitors of GABAA (bicuculline) and AMPA (DNQX) receptors. Although DNQX may also partially inhibit NMDARs (Pellegrini-Giampietro et al., 1989; Patel et al., 1990; Rao et al., 1990; Kubicki et al., 1996), it should not change the shape of I-V curve and, therefore, it does not affect the estimation of conductances.

The reported alteration in the AMPA/NMDA peak conductance ratio can be equally attributed to the augmentation of AMPAR-mediated conductance and the reduction of NMDAR-mediated conductance. Recent findings indicate that the first option is most likely. During SE, AMPAR-mediated transmission is enhanced (Abegg et al., 2004; Rakhade et al., 2008; Joshi et al., 2017). Our measurements of the I/O curve slope for fEPSP recorded in CA1 also suggest that AMPAR conductance increases on the first day following SE.

It is assumed that the enhanced synaptic AMPAR-mediated transmission is in part due to the increased surface expression of the GluA1 subunit (Abegg et al., 2004; Joshi et al., 2017) and some posttranslational modifications of the AMPARs (Rakhade et al., 2008). In our previous study, we also found a similar increase in GluA1-containing AMPARs in medial prefrontal cortex pyramidal neurons following PILO-induced SE (Malkin et al., 2016). Electrophysiological data obtained with the use of different models of epilepsy also suggest the insertion of GluA2-lacking calcium-permeable AMPARs in synapses of pyramidal cells after epileptic seizures (Prince et al., 2000; Sanchez et al., 2001; Rajasekaran et al., 2012; Joshi et al., 2017). The transient expression of such receptors may be an important element of the seizure-induced hyperexcitability that constitutes epileptogenesis. It could sustain SE by increasing neuronal synchronization and could thus facilitate the spread of seizures (Joshi et al., 2017). Although we did not investigate if Pilo-treated rats developed spontaneous recurrent seizures, according to the previously published ontogenic studies of a lithium-pilocarpine model of epilepsy, most of 21 day-old rats injected with lithium–pilocarpine became epileptic (Hirsch et al., 1992; Dube et al., 2001). Expression of these receptors prolongs elevation of internal calcium concentration levels, which can contribute to the epileptogenesis through the expression of some calcium-modulated transcription factors (Raza et al., 2004). The insertion of calcium-permeable AMPARs after seizures can also provoke the delayed neurodegeneration (Pellegrini-Giampietro et al., 1997; Grooms et al., 2000), which has been reported in different animal models (Curia et al., 2014). In line with the suggestion that enhanced excitability in AMPARs might be a reason for neurodegeneration, our finding is that the AMPA/NMDA peak conductance ratio in TC pyramidal neurons does not change after SE. In a lithium-pilocarpine model of epilepsy, the histopathological alterations are more severe in the hippocampus than in neocortical areas (Curia et al., 2008).

The enhanced excitability in AMPARs and the trafficking of the GluA1 subunit resembles the increase in synaptic efficacy observed in long-term potentiation (LTP) (Nicoll and Malenka, 1999; Collingridge et al., 2004; Plant et al., 2006). Some experimental studies support the suggestion that epileptic activity may potentiate the glutamatergic synapses similar to LTP induction protocols by activation of the same pathway (Abegg et al., 2004; Debanne et al., 2006). For example, the synaptic strengthening induced by epileptic activity in slices was prevented by NMDAR blockade during bursting (Ben-Ari and Gho, 1988; Abegg et al., 2004). Overnight epileptiform activity in slices prevented LTP induction by theta-burst stimulation, suggesting that synapses were already maximally potentiated; the occlusion of synaptic potentiation was accompanied by an increase in long-term synaptic depression (Abegg et al., 2004). Similar impairments of LTP have been observed in a kindling model of epilepsy (Schubert et al., 2005; Cunha et al., 2015), after PTZ-induced SE (Postnikova et al., 2017), and after PILO-induced SE (Kryukov et al., 2016). Homeostatic mechanisms quickly rein in synaptic efficacy close to the baseline, thereby preserving the ability of synapses to undergo LTP (Seeburg and Sheng, 2008). Indeed, we found that on the third day, the AMPA/NMDA peak conductance ratio in CA1 pyramidal neurons returns to the control value and the slope of the I/O curve for fEPSP recorded in CA1 does not differ from that of control animals. Thus, the PILO-induced SE transiently increases AMPAR-mediated neurotransmission of CA1 pyramidal neurons. These changes in neurotransmission could be an important component of the cascade of events within epileptogenesis.

### Changes in NMDAR subunit composition following SE and functional significance of findings

Using RT-PCR and electrophysiological techniques, we found that PILO-induced acute seizures increase the proportion of GluN2B-containing NMDARs in hippocampal pyramidal cell synapses, but do not change the properties of NMDARs in the TC. Similar shifts in the production of individual subunits of NMDARs following acute seizures have been shown in several experimental models. Early upregulation of GluN2B subunit production is found in the hippocampus in PTZ (Postnikova et al., 2017), PTZ-kindling (Zhu et al., 2015), and PILO (Di Maio et al., 2011, 2013) models. Di Maio et al. (2013) found that in rat primary hippocampal cultures, PILO exposure induced overexpression of GluN2A and GluN2B subunits. Notably, GluN2B overexpression plays a crucial role in triggering neuronal hyperexcitability (Di Maio et al., 2013). Contradictory data about NMDAR subunit expression were obtained in other experimental models. A significant reduction in the production of the GluN2B subunit in various regions of the hippocampus and cerebellum and an increase in the expression of the GluN2A subunit are revealed after repetitive seizures, induced by repeated administration (4–7 days) of convulsant drug 3-mercaptopropionic acid (Auzmendi et al., 2009; Girardi et al., 2010; Gori and Girardi, 2013). A significant increase of GluN2A, but not of GluN2B expression in the immunohistochemical analysis was obtained after repeated epileptic seizures induced for 12 days by intraperitoneal administration of 4-aminopyridine (Borbely et al., 2009). The difference between models suggests that seizures may activate independent pathways that are critical for aberrant NMDAR subunit expression.

Importantly, the upregulation of GluN2B subunit production is observed in the hippocampus of rats with experimental epilepsy (Colciaghi et al., 2011; Klatte et al., 2013; Muller et al., 2013; Peng et al., 2016). Some studies have shown that NMDARs with a high proportion of GluN2B subunits appear upregulated in epileptogenic tissue from patients with epilepsy and SE (Mathern et al., 1998; Loddenkemper et al., 2014), focal cortical dysplasia (Crino et al., 2001; Moddel et al., 2005; Finardi et al., 2006),and tuberous sclerosis complex (Talos et al., 2008). However, the downregulation of GluN2B in human epileptic patients affected by periventricular nodular heterotopia, subcortical band heterotopia has also been reported (Finardi et al., 2006).

The subunit composition determines the NMDAR properties, sign of synaptic plasticity, and neuronal mechanisms implicated in neuropsychiatric disorders (Paoletti et al., 2013). Therefore, changes in subunit composition have some significant consequences. First, the observed changes in subunit composition are associated with slower NMDAR-mediated EPSP kinetics are in line with the slower kinetics of GluN2B-containing NMDARs (Erreger et al., 2005; Sun et al., 2017). Because the GluN2A-dominant allosteric interaction forces the triheteromeric GluN1/2A/2B receptor into a “GluN1/2A-like” state and significantly shapes the amplitude and duration of current flux (Sun et al., 2017), we suggest that seizure induces the appearance of functional diheteromeric GluN1/2B NMDARs and decreases the proportion of GluN1/2A/2B receptors. The triheteromeric NMDAR is likely crucial for reliable adult brain function (Sun et al., 2017). In GluN2A knock-out (KO) mice, altered synaptic plasticity and disrupted learning have been demonstrated (Sakimura et al., 1995). Consistent with this, in our previous studies of this epilepsy model, we observed alterations in both LTP and long-term depression in post-SE tissue (Kryukov et al., 2016; Ivanov and Zaitsev, 2017), and impairment of exploratory behavior and spatial memory in adolescent rats (Kalemenev et al., 2015).

## Conclusion

In summary, we have provided evidence from the PILO-model in rats that acute SE leads to stronger alterations in excitatory synaptic transmission in the dorsal hippocampus than in the TC during the first 3 days following seizures. Seizures affect the relative contribution of AMPA and NMDA receptor conductances in the synaptic response and increase the proportion of GluN2B-containing NMDARs in CA1 pyramidal neurons. These alterations disturb normal circuitry functions in the hippocampus, may cause neuron damage, and may be one of the important pathogenic mechanisms of TLE. Our findings provide a solid rationale for targeting GluN2B-containing NMDARs that are specifically upregulated after acute SE to prevent epileptogenesis.

## Author contributions

DA, OZ, and AZ designed the study. DA, SM, JE, KK, EV, and OZ performed experiments and analyzed data. DA, SM, JE, KK, EV, OZ, and AZ made interpretation of data for the work. DA, OZ, and AZ wrote the manuscript. DA, SM, JE, KK, EV, OZ, and AZ approved the final version.

### Conflict of interest statement

The authors declare that the research was conducted in the absence of any commercial or financial relationships that could be construed as a potential conflict of interest.
